# Comparison between clinical significance of height-adjusted and weight-adjusted appendicular skeletal muscle mass

**DOI:** 10.1186/s40101-017-0130-1

**Published:** 2017-02-13

**Authors:** Taishi Furushima, Motohiko Miyachi, Motoyuki Iemitsu, Haruka Murakami, Hiroshi Kawano, Yuko Gando, Ryoko Kawakami, Kiyoshi Sanada

**Affiliations:** 1 College of Sport and Health Science, Ritsumeikan University, 1-1-1 Noji Higashi, Kusatsu, Shiga 525-8577 Japan; 2 Department of Health Promotion and Exercise, National Institute of Health and Nutrition, 1-23-1 Toyama, Shinjuku-ku, Tokyo, 162-8636 Japan; 3 Faculty of Letters, Kokushikan University, 4-28-1, Setagaya, Setagaya-ku, Tokyo, 154-8515 Japan; 4 Faculty of Sport Sciences, Waseda University, 2-579-15, Mikajima, Tokorozawa, Saitama 359-1192 Japan

**Keywords:** Sarcopenia, Definition, Body height, Body weight, Cardiometabolic diseases, Osteoporosis

## Abstract

**Background:**

This study aimed to compare relationships between height- or weight-adjusted appendicular skeletal muscle mass (ASM/Ht^2^ or ASM/Wt) and risk factors for cardiometabolic diseases or osteoporosis in Japanese men and women.

**Methods:**

Subjects were healthy Japanese men (*n =* 583) and women (*n =* 1218). The study population included a young group (310 men and 357 women; age, 18–40 years) and a middle-aged and elderly group (273 men and 861 women; age, ≥41 years). ASM was measured by dual-energy X-ray absorptiometry. The reference values for class 1 and 2 sarcopenia in each sex were defined as values one and two standard deviations below the sex-specific means of the young group, respectively.

**Results:**

The reference values for class 1 and 2 sarcopenia defined by ASM/Ht^2^ were 7.77 and 6.89 kg/m^2^ in men and 6.06 and 5.31 kg/m^2^ in women, respectively. The reference values for ASM/Wt were 35.0 and 32.0% in men and 29.6 and 26.4% in women, respectively. In both men and women, ASM/Wt was negatively correlated with higher triglycerides (TG) and positively correlated with serum high-density lipoprotein cholesterol (HDL-C), but these associations were not found in height-adjusted ASM. In women, TG, systolic blood pressure, and diastolic blood pressure in sarcopenia defined by ASM/Wt were significantly higher than those in normal subjects, but these associations were not found in sarcopenia defined by ASM/Ht^2^. Whole-body and regional bone mineral density in sarcopenia defined by ASM/Ht^2^ were significantly lower than those in normal subjects, but these associations were not found in sarcopenia defined by ASM/Wt.

**Conclusions:**

Weight-adjusted definition was able to identify cardiometabolic risk factors such as TG and HDL-C while height-adjusted definition could identify factors for osteoporosis.

## Background

“Sarcopenia” refers to a reduction in muscle mass and function with age [1] and leads to physical disabilities [2, 3], falls [2], and osteoporosis [4]. Sarcopenia also increases the risk of developing chronic disorders, including cardiovascular diseases (CVD) [5] and type 2 diabetes [6].

Although sarcopenia is often defined by relative muscle mass, different methods for normalizing values, such as by adjusting for height (Ht) and body weight (Wt), have been widely reported. Baumgartner et al. previously defined sarcopenia by height-adjusted appendicular skeletal muscle mass (ASM/Ht^2^, kg/m^2^) in a study involving Hispanic and non-Hispanic white men and women [2]. They reported that sarcopenia was significantly associated with self-reported physical disability in both sexes, independently of ethnicity, age, morbidity, obesity, income, and hearth condition. In contrast, Janssen et al. defined sarcopenia by weight-adjusted ASM (ASM/Wt × 100, %) in a study of non-Hispanic white people, non-Hispanic black people, and Mexican Americans [7]. They also reported that the age-related decrease of relative muscle mass is significantly and independently associated with functional impairment and disability, particularly in older women.

With regard to Asian populations, Hong et al. investigated the association of sarcopenia defined by height-adjusted ASM and sites of fragility fractures in Chinese elderly [8]. Logistic regression analyses in that study revealed that lower height-adjusted ASM was associated with an increased risk of hip fracture both in men and women. In elderly Korean people, height-adjusted ASM was positively correlated with bone mineral density (BMD), and a strong correlation was found between sarcopenia and osteoporosis with risk of bone fracture [9].

Park et al. reported that CVD prevalence was positively associated with sarcopenia defined by weight-adjusted ASM in men after adjusting for CVD risk factors, but no such association was found in women [10]. Similarly, Byeon et al. reported that in a ≥60-year-old Korean study population, sarcopenic individuals defined by weight-adjusted ASM had a higher Framingham risk score than non-sarcopenic individuals in a non-obese group [11]. Moreover, in a Thai population, Pongchaiyakul et al. found that an urban environment is the strongest predictive factor for sarcopenia defined by weight-adjusted ASM, followed by high body mass index (BMI) and age [12]. These previous studies suggest that the relationship between sarcopenia and the risk of lifestyle-related diseases such as cardiometabolic diseases and osteoporosis depends on the method used to normalize muscle mass in Asian populations.

Lim et al. previously compared risk factors for metabolic syndrome in Korean individuals with sarcopenia defined by height-adjusted and weight-adjusted ASM [13]. In that study, however, the sex-specific young reference group used to determine cut-off points for sarcopenia had a small number of subjects.

Although Sanada et al. reported reference values for height-adjusted ASM in sarcopenia for young Japanese adult men and women [14], it is unclear whether different relationships would result between sarcopenia defined by height-adjusted ASM or weight-adjusted ASM and risk factors for cardiometabolic diseases or osteoporosis in Japanese populations.

Against this backdrop, this study aimed to compare relationships between height- or weight-adjusted ASM and risk factors for cardiometabolic diseases or osteoporosis in Japanese men and women.

## Methods

### Subjects

Subjects were healthy Japanese men (*n =* 583) and women (*n =* 1218) aged 18–90 years. The study population included a young group (310 men and 357 women; age, 18–40 years) which served as a basis to define the reference values referred by Baumgartner et al. [2], and a middle-aged and elderly group (273 men and 861 women; age, ≥41 years). The subjects were recruited from people participating in a Nutrition and Exercise Intervention Study (NEXIS, registered at ClinicalTrials.gov, Identifier: NCT00926744). We excluded men and women with obesity (BMI ≥25), CVD, and stroke, as determined using a medical history questionnaire. They were sedentary or moderately active and participated in a swimming, stretching, and “healthy gymnastics” program; however, none of the subjects participated in other vigorous sports activities. The purpose, procedures, and risks of the study were explained to all participants prior to inclusion, and all subjects gave their written informed consent before enrolling in the study. The study was performed in accordance with the guidelines of the Declaration of Helsinki and was approved by the Human Research Committee of the National Institute of Health and Nutrition, Tokyo, Japan (KENEI14-02). Body weight and height were recorded, and BMI was calculated as weight (kg) divided by the square of the height (m). Waist circumference (WC) was measured at the uppermost border of the iliac crests.

### Analysis of blood samples

Blood was drawn from subjects in the seated position. Fasting (>12 h) blood samples were collected by venipuncture in tubes with or without ethylene diamine tetraacetic acid (for plasma or serum). Blood samples were centrifuged at 1500 rpm for 15 min and stored at –20 °C. Serum concentration of triglycerides (TG) was determined using commercial kits (Mitsubishi Chemical Medience, Tokyo, Japan). Serum high-density lipoprotein cholesterol (HDL-C) was measured by an enzymatic method (Mitsubishi Chemical Medience). Whole-blood glycohemoglobin A1c (HbA1c) was measured by an enzymatic method (glycohemoglobin A1c kit; Mitsubishi Chemical Medience).

### Analysis of arterial blood pressure at rest

Systolic blood pressure (SBP) and diastolic blood pressure (DBP) were measured at rest using a vascular testing device (Colin Medical Technology, Tokyo, Japan). Chronic arterial blood pressure levels at rest were measured with the same device over the brachial and dorsalis pedis arteries. Recordings were made in triplicate with subjects in the supine position. Brachial-ankle pulse wave velocity (baPWV), which provides qualitatively similar information to that derived from central arterial stiffness, was measured by the volume plethysmographic method.

### Whole-body dual-energy X-ray absorptiometry

Lean soft tissue mass and BMD were determined by whole-body dual-energy X-ray absorptiometry (DXA) (Hologic QDR-4500A scanner; Hologic, Waltham, MA). Whole-body lean soft tissue mass was divided into several regions, i.e., the arms, legs, and trunk. Body composition was determined by Hologic software version 11.2:3 for Windows (Hologic, Waltham, MA). The reference values (ASM/Ht^2^, ASM/Wt × 100) for class 1 and class 2 sarcopenia in each sex were defined as values one and two standard deviations (SD) below the sex-specific means of the reference data for young adults aged 18–40 years, respectively.

### Measures of fitness

Handgrip strength of the right upper limb was measured using a handheld dynamometer. In the standing position, with the arm straight by the side, the subjects gripped the dynamometer as hard as possible for 3 s without pressing the instrument against the body or bending at the elbow. Value was recorded as the average of two trials.

### Statistical analysis

All measurements and calculated values are expressed as mean ± SD. Partial correlations between height-adjusted ASM and weight-adjusted ASM were analyzed after adjusting for age and percent body fat. Differences between prevalence of sarcopenia defined by height-adjusted ASM and weight-adjusted ASM were assessed by the chi-square test. We compared mean values of physical characteristics, body composition, fitness, and risk factors for cardiometabolic diseases between normal, class 1 and class 2 sarcopenia by one-way ANCOVA after adjusting for age and percent body fat. Student’s unpaired *t* test was used to test differences among normal, class 1 and class 2 sarcopenic subjects. The alpha level for testing significance was set at *P* < 0.05. All statistical analyses were performed using StatView version 5.0 for Window (SAS Institute, Cary, NC, USA).

## Results

The physical characteristics of young group and middle-aged and elderly group are shown in Table 1. Height-adjusted ASM in young men and women aged 18–40 years were 8.66 ± 0.89 kg/m^2^ and 6.80 ± 0.75 kg/m^2^, respectively. Weight-adjusted ASM in young men and women were 38.1 ± 3.0% and 32.7 ± 3.2%, respectively. Therefore, in this study, the cut-off values for class 1 sarcopenia using height-adjusted ASM in men and women were set as 7.77 and 6.06 kg/m^2^ and the cut-off values for class 2 sarcopenia were set as 6.89 and 5.31 kg/m^2^, respectively. In addition, the cut-off values for class 1 sarcopenia using weight-adjusted ASM in men and women were set as 35.0 and 29.6% and the cut-off values for class 2 sarcopenia were set as 32.0 and 26.4%, respectively. In the middle-aged and elderly group aged 41–90 years, height-adjusted ASM in men and women were 8.05 ± 0.68 kg/m^2^ and 6.47 ± 0.58 kg/m^2^, respectively. Weight-adjusted ASM in men and women were 35.9 ± 2.6% and 30.3 ± 2.9%, respectively.

**Table 1:** Physical characteristics of young group and middle-aged and elderly group

	Young group		Middle-aged and elderly group	
	Men	Women	Men	Women
Number	310	357	273	861
Age (years)	29 ± 7	29 ± 7	60 ± 12	60 ± 11
Height (cm)	173.2 ± 5.6	160.4 ± 5.9	168.7 ± 6.3	155.7 ± 5.8
Weight (kg)	68.6 ± 8.8	53.9 ± 7.2	63.9 ± 6.7	52.0 ± 5.8
BMI (kg/m^2^)	22.9 ± 2.8	20.9 ± 2.5	22.4 ± 1.5	21.4 ± 1.9
WC (cm)	79.2 ± 7.9	73.0 ± 8.4	82.1 ± 5.6	79.6 ± 7.2
Percent body fat (%)	16.7 ± 4.8	24.4 ± 5.7	20.0 ± 3.9	28.0 ± 5.2
Handgrip strength (kg)	44.5 ± 7.1	29.3 ± 5.3	38.6 ± 7.3	25.1 ± 4.5
ASM/Ht^2^ (kg/m^2^)	8.66 ± 0.89	6.80 ± 0.75	8.05 ± 0.68	6.47 ± 0.58
ASM/Wt × 100 (%)	38.1 ± 3.0	32.7 ± 3.2	35.9 ± 2.6	30.3 ± 2.9

Data are presented as mean ± SD

*BMI* body mass index, *WC* waist circumference, *ASM* appendicular skeletal muscle mass, *Ht* height, *Wt* weight

Partial correlation coefficients adjusted for age are shown in Table 2. After adjusting for age, partial correlation coefficients between height-adjusted ASM and weight-adjusted ASM were 0.577 in men (*P <* 0.001) and 0.467 in women (*P <* 0.001), and Wt, BMI, and WC were positively correlated with height-adjusted ASM but negatively correlated with weight-adjusted ASM in men and women (*P <* 0.01). HbA1c, TG, SBP, and DBP were negatively, and HDL-C was positively, correlated with weight-adjusted ASM (*P <* 0.01), but not with height-adjusted ASM in women. In men, lumbar spine BMD was positively correlated with ASM/Ht^2^ (*P <* 0.05), but not with ASM/Wt. ASM/Ht^2^ had higher correlation coefficients with whole-body, arm, lumber spine, and leg BMD than ASM/Wt in both men and women. In men and women, baPWV was negatively correlated with both ASM/Ht^2^ and ASM/Wt (*P <* 0.05).

**Table 2:** Partial correlation coefficients adjusted for age between ASM and measured values in middle-aged and elderly men and women

	Men				Women			
	ASM/Ht^2^ (kg/m^2^)		ASM/Wt × 100 (%)		ASM/Ht^2^ (kg/m^2^)		ASM/Wt × 100 (%)	
	*R*	*P* value	*R*	*P* value	*R*	*P* value	*R*	*P* value
ASM/Ht^2^ (kg/m^2^)	–	–	0.577	<0.001	–	–	0.467	<0.001
ASM/Wt × 100 (%)	0.577	<0.001	–	–	0.467	<0.001	–	–
Height (cm)	0.086	N.S.	0.097	<0.01	0.039	N.S.	0.148	<0.001
Weight (kg)	0.427	<0.001	−0.199	<0.01	0.431	<0.001	−0.373	<0.001
BMI (kg/m^2^)	0.538	<0.001	−0.374	<0.001	0.478	<0.001	−0.549	<0.001
WC (cm)	0.198	<0.01	−0.485	<0.001	0.209	<0.001	−0.558	<0.001
Percent body fat (%)	−0.239	<0.001	−0.729	<0.001	−0.251	<0.001	−0.896	<0.001
Handgrip strength (kg)	0.383	<0.001	0.394	<0.001	0.340	<0.001	0.283	<0.001
Whole-body BMD (g/cm^2^)	0.352	<0.001	0.139	<0.05	0.275	<0.001	0.207	<0.001
Arm BMD (g/cm^2^)	0.422	<0.001	0.190	<0.01	0.324	<0.001	0.249	<0.001
Lumbar spine BMD (g/cm^2^)	0.206	<0.05	0.080	N.S.	0.244	<0.001	0.150	<0.001
Leg BMD (g/cm^2^)	0.442	<0.001	0.167	<0.01	0.378	<0.001	0.174	<0.001
HbA1c (%)	−0.032	N.S.	0.019	N.S.	0.009	N.S.	−0.104	<0.01
TG (mg/dl)	−0.032	N.S.	−0.153	<0.05	−0.045	N.S.	−0.224	<0.001
HDL-C (mg/dl)	0.051	N.S.	0.246	<0.001	0.001	N.S.	0.333	<0.001
TG/HDL ratio	−0.033	N.S.	−0.179	<0.05	−0.039	N.S.	−0.268	<0.001
SBP (mmHg)	0.060	N.S.	−0.011	N.S.	0.035	N.S.	−0.114	<0.01
DBP (mmHg)	0.141	<0.05	−0.039	N.S.	0.034	N.S.	−0.127	<0.001
baPWV (cm/s)	−0.214	<0.01	−0.149	<0.05	−0.153	<0.001	−0.141	<0.001

*ASM* appendicular skeletal muscle mass, *Ht* height, *Wt* weight, *BMI* body mass index, *WC* waist circumference, *BMD* bone mineral density, *H﻿bA1c* ﻿glycohemoglobin﻿ A1c, *TG* triglycerides, *HDL-C* high-density lipoprotein cholesterol, *SBP* systolic blood pressure, *DBP* diastolic blood pressure, *baPWV* brachial-ankle pulse wave velocity

Partial correlation coefficients adjusted for age and percent body fat are shown in Table 3. After adjusting for age and percent body fat, partial correlation coefficients between height-adjusted ASM and weight-adjusted ASM were 0.630 in men (*P <* 0.001) and 0.577 in women (*P <* 0.001). HbA1c, TG, and TG/HDL-C were not significantly correlated with ASM/Ht^2^ and ASM/Wt in both men and women. In men, whole-body, arm, lumbar spine, and leg BMD were positively correlated with ASM/Ht^2^ (*P <* 0.05), but not with ASM/Wt. In women, whole-body and lumbar spine BMD were positively correlated with ASM/Ht^2^ (*P <* 0.001), but not with ASM/Wt. In men and women, baPWV was negatively correlated with both ASM/Ht^2^ and ASM/Wt (*P <* 0.001).

**Table 3:** Partial correlation coefficients adjusted for age and percent body fat between ASM and measured values in middle-aged and elderly men and women

	Men				Women			
	ASM/Ht^2^ (kg/m^2^)		ASM/Wt × 100 (%)		ASM/Ht^2^ (kg/m^2^)		ASM/Wt × 100 (%)	
	*R*	*P* value	*R*	*P* value	*R*	*P* value	*R*	*P* value
ASM/Ht^2^ (kg/m^2^)	–	–	0.630	<0.001	–	–	0.577	<0.001
ASM/Wt × 100 (%)	0.630	<0.001	–	–	0.577	<0.001	–	–
Height (cm)	0.036	N.S.	0.166	<0.05	0.029	N.S.	0.250	<0.001
Weight (kg)	0.605	<0.001	0.266	<0.001	0.679	<0.001	0.277	<0.001
BMI (kg/m^2^)	0.889	<0.001	0.211	<0.01	0.870	<0.001	0.113	<0.01
WC (cm)	0.499	<0.001	0.162	<0.05	0.477	<0.001	0.092	<0.05
Handgrip strength (kg)	0.287	<0.001	0.188	<0.01	0.278	<0.001	0.167	<0.001
Whole-body BMD (g/cm^2^)	0.306	<0.001	−0.026	N.S.	0.217	<0.001	0.028	N.S.
Arm BMD (g/cm^2^)	0.366	<0.001	0.041	N.S.	0.282	<0.001	0.171	<0.001
Lumbar spine BMD (g/cm^2^)	0.150	<0.05	−0.137	<0.05	0.206	<0.001	−0.018	N.S.
Leg BMD (g/cm^2^)	0.414	<0.001	0.082	N.S.	0.360	<0.001	0.203	<0.001
HbA1c (%)	−0.040	N.S.	−0.031	N.S.	0.056	N.S.	0.000	N.S.
TG (mg/dl)	−0.033	N.S.	0.012	N.S.	0.031	N.S.	−0.003	N.S.
HDL-C (mg/dl)	−0.003	N.S.	−0.114	N.S.	−0.093	<0.05	0.036	N.S.
TG/HDL ratio	−0.018	N.S.	0.060	N.S.	0.046	N.S.	−0.024	N.S.
SBP (mmHg)	0.068	N.S.	−0.069	N.S.	0.077	<0.05	−0.057	N.S.
DBP (mmHg)	0.157	<0.05	0.007	N.S.	0.073	<0.05	−0.087	<0.05
baPWV (cm/s)	−0.240	<0.001	−0.241	<0.001	−0.133	<0.001	−0.152	<0.001

*ASM* appendicular skeletal muscle mass, *Ht* height, *Wt* weight, *BMI* body mass index, *WC* waist circumference, *BMD* bone mineral density, *H﻿bA1c* glycoh﻿emoglobin A1c, *TG* triglycerides, *HDL-C* high-density lipoprotein cholesterol, *SBP* systolic blood pressure, *DBP* diastolic blood pressure, *baPWV* brachial-ankle pulse wave velocity

When sarcopenia was defined by weight-adjusted ASM, the prevalence rates of class 1 and class 2 sarcopenia in the subjects aged 41–90 years were 32.6% (89/273) and 5.9% (16/273) in men (Table 4) and 35.5% (306/861) and 7.1% (61/861) in women (Table 5), respectively. When defined by height-adjusted ASM, the prevalence rates of class 1 and class 2 sarcopenia in the subjects aged 41–90 years were 27.8% (76/273) and 4.4% (12/273) in men (Table 4) and 22.3% (192/861) and 1.5% (13/861) in women (Table 5), respectively. The prevalence rates of sarcopenia significantly differed when defined by height-adjusted ASM and weight-adjusted ASM in both men and women (*P <* 0.001; chi-square test). In the subjects aged 65–90 years, the prevalence rates of class 1 and class 2 sarcopenia defined by weight-adjusted ASM were 39.8% (43/108) and 10.2% (11/108) in men, and 39.8% (125/314) and 9.2% (29/314) in women, respectively. When defined by height-adjusted ASM, the prevalence rates of class 1 and class 2 sarcopenia were 39.8% (43/108) and 9.3% (10/108) in men and 24.2% (76/314) and 2.5% (8/314) in women, respectively.

**Table 4:** Characteristics of middle-aged and elderly men with sarcopenia defined by height-adjusted ASM or weight-adjusted ASM

	Sarcopenia defined by ASM/Ht^2^					Sarcopenia defined by ASM/Wt				
	Normal	Class 1	Class 2	*P value		Normal	Class 1	Class 2	*P value	
		Sarcopenia	Sarcopenia	One-way ANCOVA	Post hoc analysis		Sarcopenia	Sarcopenia	One-way ANCOVA	Post hoc analysis
Number	185 (67.8%)	76 (27.8%)	12 (4.4%)	–	–	168 (61.5%)	89 (32.6%)	16 (5.9%)	–	–
Age (years)	57 ± 10	64 ± 13	70 ± 14	–	–	57 ± 11	62 ± 12	69 ± 11	–	–
Height (cm)	169.5 ± 6.2	167.5 ± 5.9	164.4 ± 7.4	N.S.		169.2 ± 6.5	168.1 ± 5.8	166.6 ± 6.7	N.S.	
Weight (kg)	66.0 ± 5.5	60.5 ± 6.3	53.4 ± 6.5	<0.001	a,b,c	63.5 ± 6.8	64.8 ± 6.1	63.1 ± 8.2	<0.01	
BMI (kg/m^2^)	23.0 ± 1.2	21.5 ± 1.5	19.5 ± 1.4	<0.001	a,b,c	22.1 ± 1.6	22.9 ± 1.3	22.7 ± 1.8	N.S.	
WC (cm)	82.9 ± 5.2	81.5 ± 5.8	75.1 ± 5.2	<0.001	a,b,c	80.2 ± 5.1	85.1 ± 4.4	84.9 ± 7.3	N.S.	
Percent body fat (%)	19.7 ± 3.7	20.8 ± 4.2	20.1 ± 4.0	–	–	18.1 ± 3.4	22.9 ± 2.0	24.4 ± 3.4	–	–
Handgrip strength (kg)	40.5 ± 7.0	34.9 ± 6.1	31.9 ± 6.5	<0.001	a,b	40.7 ± 6.9	35.5 ± 6.7	33.5 ± 6.1	<0.01	a,b
Whole-body BMD (g/cm^2^)	1.142 ± 0.109	1.078 ± 0.084	1.019 ± 0.113	<0.001	a,b	1.133 ± 0.104	1.097 ± 0.107	1.091 ± 0.135	N.S.	
Arm BMD (g/cm^2^)	0.799 ± 0.059	0.752 ± 0.049	0.713 ± 0.057	<0.001	a,b,c	0.793 ± 0.059	0.766 ± 0.061	0.755 ± 0.061	N.S.	
Lumbar spine BMD (g/cm^2^)	1.076 ± 0.190	1.012 ± 0.193	0.944 ± 0.223	<0.01	a,b	1.065 ± 0.181	1.027 ± 0.206	1.067 ± 0.261	N.S.	
Leg BMD (g/cm^2^)	1.246 ± 0.113	1.158 ± 0.088	1.068 ± 0.089	<0.001	a,b,c	1.234 ± 0.117	1.184 ± 0.103	1.176 ± 0.140	N.S.	
HbA1c (%)	5.29 ± 0.54	5.45 ± 0.63	5.85 ± 1.11	N.S.		5.32 ± 0.60	5.36 ± 0.54	5.76 ± 0.88	N.S.	
TG (mg/dl)	104.2 ± 52.5	100.7 ± 51.3	109.4 ± 46.22	N.S.		96.8 ± 47.0	115.1 ± 59.8	109.5 ± 41.0	N.S.	
HDL-C (mg/dl)	59.7 ± 13.7	61.4 ± 15.1	65.1 ± 17.0	N.S.		62.1 ± 14.5	57.4 ± 13.2	59.0 ± 15.9	N.S.	
TG/HDL ratio	1.92 ± 1.20	1.83 ± 1.23	1.81 ± 0.86	N.S.		1.73 ± 1.12	2.18 ± 1.33	1.92 ± 0.71	N.S.	
SBP (mmHg)	126 ± 14	126 ± 16	127 ± 23	N.S.		125 ± 14	127 ± 15	132 ± 18	N.S.	
DBP (mmHg)	80 ± 10	77 ± 11	74 ± 10	<0.05	a	78 ± 10	79 ± 10	82 ± 11	N.S.	
baPWV (cm/s)	1402 ± 247	1529 ± 308	1758 ± 399	N.S.		1388 ± 243	1515 ± 278	1773 ± 436	<0.001	a,b,c

Data are presented as mean ± SD

*BMI* body mass index, *WC* waist circumference, *BMD* bone mineral density, *H﻿b﻿A1c* glycohemoglobin A1c, *TG* triglycerides, *HDL-C* high-density lipoprotein cholesterol, *SBP* systolic blood pressure, *DBP* diastolic blood pressure, *baPWV* brachial-ankle pulse wave velocity

*One-way ANCOVA adjusted for age and percent body fat and post hoc analysis using the least significant difference *t* test (mean difference between two groups): a, Normal vs. class 1; b, Normal vs. class 2; c, class 1 vs. class 2; all *P <* 0.05

**Table 5:** Characteristics of middle-aged and elderly women with sarcopenia defined by height-adjusted ASM or weight-adjusted ASM

	Sarcopenia defined by ASM/Ht^2^					Sarcopenia defined by ASM/Wt				
	Normal	Class 1	Class 2	*P value		Normal	Class 1	Class 2	*P value	
		Sarcopenia	Sarcopenia	One-way ANCOVA	Post hoc analysis		Sarcopenia	Sarcopenia	One-way ANCOVA	Post hoc analysis
Number	656 (76.2%)	192 (22.3%)	13 (1.5%)	–	–	494 (57.4%)	306 (35.5%)	61 (7.1%)	–	–
Age (years)	60 ± 11	62 ± 11	67 ± 11	–	–	59 ± 11	62 ± 10	64 ± 10	–	–
Height (cm)	155.9 ± 5.9	155.3 ± 5.7	152.8 ± 5.8	N.S.		156.6 ± 5.9	154.6 ± 5.6	153.7 ± 5.7	<0.01	a,b
Weight (kg)	53.1 ± 5.5	48.6 ± 5.0	44.4 ± 5.5	<0.001	a,b,c	50.8 ± 5.8	53.3 ± 5.5	55.1 ± 4.8	<0.001	a,b,c
BMI (kg/m^2^)	21.9 ± 1.8	20.1 ± 1.8	18.9 ± 1.9	<0.001	a,b,c	20.7 ± 1.8	22.3 ± 1.6	23.3 ± 1.3	N.S.	
WC (cm)	80.4 ± 6.9	77.5 ± 7.3	74.7 ± 7.3	<0.001	a,b,c	76.7 ± 6.3	82.7 ± 6.1	87.1 ± 6.5	N.S.	
Percent body fat (%)	27.5 ± 5.2	29.3 ± 5.0	30.6 ± 5.4	–	–	24.8 ± 4.1	31.4 ± 2.6	36.1 ± 2.4	–	–
Handgrip strength (kg)	25.7 ± 4.5	23.3 ± 3.7	19.7 ± 4.3	<0.001	a,b,c	26.1 ± 4.6	23.9 ± 3.8	22.4 ± 4.1	N.S.	
Whole-body BMD (g/cm^2^)	1.003 ± 0.117	0.956 ± 0.102	0.883 ± 0.112	<0.001	a,b,c	1.012 ± 0.119	0.965 ± 0.106	0.945 ± 0.093	N.S.	
Arm BMD (g/cm^2^)	0.633 ± 0.067	0.605 ± 0.058	0.561 ± 0.064	<0.001	a,b,c	0.640 ± 0.070	0.609 ± 0.057	0.598 ± 0.050	N.S.	
Lumbar spine BMD (g/cm^2^)	0.952 ± 0.174	0.888 ± 0.156	0.747 ± 0.166	<0.001	a,b,c	0.961 ± 0.181	0.902 ± 0.157	0.885 ± 0.144	N.S.	
Leg BMD (g/cm^2^)	1.026 ± 0.106	0.97 ± 0.095	0.884 ± 0.104	<0.001	a,b,c	1.030 ± 0.112	0.990 ± 0.095	0.968 ± 0.082	N.S.	
HbA1c (%)	5.38 ± 0.52	5.43 ± 0.53	5.36 ± 0.52	N.S.		5.31 ± 0.41	5.48 ± 0.64	5.56 ± 0.57	N.S.	
TG (mg/dl)	82.6 ± 39.6	88.0 ± 46.1	90.2 ± 45.7	N.S.		76.0 ± 34.6	90.9 ± 44.5	112.5 ± 54.5	<0.05	a,b,c
HDL-C (mg/dl)	71.3 ± 16.8	70.2 ± 15.3	72.5 ± 15.8	N.S.		75.0 ± 16.9	66.8 ± 14.4	61.3 ± 14.3	N.S.	
TG/HDL ratio	1.29 ± 0.92	1.39 ± 1.05	1.47 ± 1.05	N.S.		1.11 ± 0.71	1.52 ± 1.11	2.00 ± 1.32	<0.05	a,b,c
SBP (mmHg)	120 ± 17	122 ± 20	120 ± 19	N.S.		118 ± 17	123 ± 18	130 ± 20	<0.05	a,b,c
DBP (mmHg)	71 ± 10	72 ± 12	69 ± 11	N.S.		70 ± 10	72 ± 10	75 ± 12	<0.05	a,b,c
baPWV (cm/s)	1319 ± 239	1433 ± 313	1607 ± 316	<0.001	a,b,c	1306 ± 245	1386 ± 270	1512 ± 301	<0.01	a,b,c

Data are presented as mean ± SD

*BMI* body mass index, *WC* waist circumference, *BMD* bone mineral density, *H﻿b﻿A1c* glycoh﻿﻿emoglobin A1c, *TG* triglycerides, *HDL-C* high-density lipoprotein cholesterol, *SBP* systolic blood pressure, *DBP* diastolic blood pressure, *baPWV* brachial-ankle pulse wave velocity

*One-way ANCOVA adjusted for age and percent body fat and post hoc analysis using the least significant difference *t* test (mean difference between two groups): a, Normal vs. class 1; b, Normal vs. class 2; c, class 1 vs. class 2; all *P <* 0.05

Health-related indexes of middle-aged and elderly men (age ≥41 years) are shown in Table 4. When sarcopenia was defined by height-adjusted ASM, BMI and WC in class 1 and class 2 sarcopenic subjects were significantly lower than those in normal subjects (*P <* 0.05), but when sarcopenia was defined by weight-adjusted ASM, BMI and WC did not significantly differ between the three groups. Whole-body BMD, arm BMD, lumbar spine BMD, and leg BMD in class 1 and 2 sarcopenic subjects defined by height-adjusted ASM were significantly lower than those in normal subjects (*P <* 0.05). When sarcopenia was defined by weight-adjusted ASM, those in class 1 and 2 sarcopenic subjects were not significantly different. When sarcopenia was defined by weight-adjusted ASM, baPWV in class 1 and 2 sarcopenic subjects was significantly higher than that in normal subjects (*P <* 0.05), but when defining sarcopenia by height-adjusted ASM, that was not significantly different.

Health-related indexes in middle-aged and elderly women are shown in Table 5. In height-adjusted definition, BMI, WC, and handgrip strength in class 1 and 2 sarcopenic subjects were significantly lower than those in normal subjects (*P <* 0.05), but not in weight-adjusted definition. When sarcopenia was defined by height-adjusted ASM, whole-body BMD, arm BMD, lumbar spine BMD, and leg BMD in class 1 and class 2 sarcopenic subjects were significantly lower than those in normal subjects (*P <* 0.05), but when sarcopenia was defined by weight-adjusted ASM, those were not significantly different. In definition of ASM/Wt, TG, TG/HDL-C, SBP, and DBP in class 1 and class 2 sarcopenic subjects were significantly higher than those in normal subjects (*P <* 0.05), but in definition of ASM/Ht^2^, those were not significantly different. In both definitions, baPWV in class 1 and 2 sarcopenic subjects was significantly higher than that in normal subjects (*P <* 0.05).

## Discussion

The major findings of our study were that height-adjusted ASM showed stronger associations with risk factors for osteoporosis than weight-adjusted ASM, and conversely, weight-adjusted ASM was more strongly associated with risk factors for cardiometabolic diseases than height-adjusted ASM.

Kim et al. previously proposed cut-off values for class 1 and class 2 sarcopenia in Korean people [15] of 32.2 and 29.1% for men and 25.6 and 23.0% for women, respectively, using the weight-adjusted definition. They also reported prevalence rates of class 1 and class 2 sarcopenia of 29.5 and 9.7% for elderly men and 30.3 and 11.8% for elderly women (age, ≥65 years), respectively, using the same definition. In this study, the prevalence rates of sarcopenia were higher than those reported in the Korean study for both sexes and our cut-off values were considerably higher because weight, BMI, and WC in our young group were lower than those in the young group from the Korean study. Moreover, absolute muscle mass in our young group was relatively higher than that reported in the Korean study.

Sanada et al. previously reported that BMI and WC in sarcopenic subjects (defined by height-adjusted ASM) were significantly lower than those in normal subjects [14]. Abe et al. also reported that, after adjusting for age, BMI, WC, and percent body fat were lower when using the height-adjusted definition [16]. This suggests that sarcopenia defined by height-adjusted ASM does not detect people at higher risk of obesity. However, Janssen et al. found that sarcopenia defined by weight-adjusted ASM was associated with higher BMI in both men and women [7]. In the present study, when sarcopenia was defined by height-adjusted ASM, WC in sarcopenic subjects was significantly lower than that in normal subjects (*P <* 0.05), but when defined by weight-adjusted ASM, WC was not significantly different for both men (Table 4) and women (Table 5). Moreover, Wt, BMI, and WC were positively correlated with height-adjusted ASM, but negatively correlated with weight-adjusted ASM after adjusting for age in both men and women (Table 2). These results suggest that sarcopenia defined by weight-adjusted ASM is associated with abdominal obesity. Thus, defining sarcopenia by weight-adjusted ASM can better detect sarcopenic individuals at higher risk of obesity than when defining sarcopenia by height-adjusted ASM.

Recently, Hamasaki et al. reported that the ratio of lower extremity muscle mass to weight measured by bioelectrical impedance was negatively correlated with BMI, WC, body fat mass, percent body fat, subcutaneous fat area, and serum-free fatty acid concentration [17]. In this study, after adjusting age, ASM/Wt was more strongly correlated with risk factors for cardiometabolic diseases than ASM/Ht^2^ (Table 2). For example, ASM/Wt was negatively correlated with TG and positively correlated with HDL-C, but ASM/Ht^2^ was not correlated with them in both men and women. ASM/Wt was also strongly correlated with percent body fat (men; *R* = −0.73, women; *R* = −0.90). Thus, it is considered that the associations between ASM/Wt and risk factors for cardiometabolic diseases were caused by negative correlations between ASM/Wt and percent body fat. After adjusting age and percent body fat, most of the significant differences were disappeared (Table 3). Therefore, it was shown that percent body fat contributed strongly to the associations between ASM/Wt and risk factors for cardiometabolic diseases. However, even after adjusting age and percent body fat, sarcopenia defined by weight-adjusted ASM in women was significantly associated with risk factors for cardiometabolic diseases, including TG, TG/HDL-C, SBP, and DBP (Table 5). On the other hand, sarcopenia defined by height-adjusted ASM was not significantly associated with them. For men, in definition of ASM/Wt, baPWV in sarcopenic subjects was significantly higher than that in normal subjects, but in definition of ASM/Ht^2^, baPWV was not significantly different (Table 4). These results suggested that even after adjusting percent body fat, sarcopenia defined by weight-adjusted ASM can detect sarcopenic individuals at higher risk of cardiometabolic diseases with more sensitivity than when defined by height-adjusted ASM.

With regard to risk factors for osteoporosis, a previous study reported that sarcopenia defined by height-adjusted ASM was associated with lower BMD in both men and women [14]. In contrast, another study reported that osteoporosis was not significantly associated with muscle mass/weight [12]. In the present study, for both men and women, whole-body BMD, arm BMD, lumbar spine BMD, and leg BMD in sarcopenic subjects (defined by height-adjusted ASM) were significantly lower than those for normal subjects. When defined by weight-adjusted ASM, however, those in sarcopenic subjects were not significantly different (Tables 4 and 5). These results suggest that sarcopenia defined by height-adjusted ASM can better detect sarcopenic individuals at higher risk of osteoporosis than when defined by weight-adjusted ASM. However, this study measured whole-body and regional BMD using only whole-body DXA. Lumbar spine BMD should be examined by the specific manner. This point is a limitation in the present study.

## Conclusions

In practice, the use of height-adjusted ASM has the advantage over the weight-adjusted ASM to detect risk factors for osteoporosis. Conversely, the use of weight-adjusted ASM has the advantage to detect risk factors for cardiometabolic diseases.

## Abbreviations

baPWV: Brachial-ankle pulse wave velocity
BMD: Bone mineral density
BMI: Body mass index
CVD: Cardiovascular diseases
DBP: Diastolic blood pressure
DXA: Dual-energy X-ray absorptiometry
HbA1c: Glycohemoglobin A1c
HDL-C: High-density lipoprotein cholesterol
Ht: Height
SBP: Systolic blood pressure
SD: Standard deviations
TG: Triglycerides
WC: Waist circumference
Wt: Body weight
